# The neurologic pain signature responds to nonsteroidal anti-inflammatory treatment vs placebo in knee osteoarthritis

**DOI:** 10.1097/PR9.0000000000000986

**Published:** 2022-02-16

**Authors:** Marina López-Solà, Jesus Pujol, Jordi Monfort, Joan Deus, Laura Blanco-Hinojo, Ben J. Harrison, Tor D. Wager

**Affiliations:** a Department of Medicine, School of Medicine and Health Sciences, Serra Hunter Faculty Program, University of Barcelona, Barcelona, Spain; b MRI Research Unit, Department of Radiology, Hospital del Mar, Barcelona, Spain; c Centro Investigación Biomédica en Red de Salud Mental, CIBERSAM, Barcelona, Spain; d Rheumatology Department, Hospital del Mar, Barcelona, Spain; e Department of Clinical and Health Psychology, Autonomous University of Barcelona, Barcelona, Spain; f Department of Psychiatry, Melbourne Neuropsychiatry Centre, The University of Melbourne & Melbourne Health, Melbourne, Australia; g Department of Psychological and Brain Sciences, Dartmouth College, Dartmouth, MA, USA

**Keywords:** Chronic pain, Osteoarthritis, fMRI, Neurologic pain signature, NSAID, Placebo

## Abstract

**Introduction:**

Many drug trials for chronic pain fail because of high placebo response rates in primary endpoints. Neurophysiological measures can help identify pain-linked pathophysiology and treatment mechanisms. They can also help guide early stop/go decisions, particularly if they respond to verum treatment but not placebo. The neurologic pain signature (NPS), an fMRI-based measure that tracks evoked pain in 40 published samples and is insensitive to placebo in healthy adults, provides a potentially useful neurophysiological measure linked to nociceptive pain.

**Objectives:**

This study aims to validate the NPS in knee osteoarthritis (OA) patients and test the effects of naproxen on this signature.

**Methods:**

In 2 studies (50 patients, 64.6 years, 75% females), we (1) test the NPS and other control signatures related to negative emotion in knee OA pain patients; (2) test the effect of placebo treatments; and (3) test the effect of naproxen, a routinely prescribed nonsteroidal anti-inflammatory drug in OA.

**Results:**

The NPS was activated during knee pain in OA (*d* = 1.51, *P* < 0.001) and did not respond to placebo (*d* = 0.12, *P* = 0.23). A single dose of naproxen reduced NPS responses (vs placebo, NPS *d* = 0.34, *P* = 0.03 and pronociceptive NPS component *d* = 0.38, *P* = 0.02). Naproxen effects were specific for the NPS and did not appear in other control signatures.

**Conclusion:**

This study provides preliminary evidence that fMRI-based measures, validated for nociceptive pain, respond to acute OA pain, do not appear sensitive to placebo, and are mild-to-moderately sensitive to naproxen.

## Abstract 2

Supplemental Digital Content is Available in the Text.

fMRI-based measures, validated for nociceptive pain, respond to acute osteoarthritis pain, are not sensitive to placebo, and are mild-to-moderately sensitive to naproxen.

## 1. Introduction

Treatment-related improvement in subjective reports of pain is usually the endpoint in clinical trials for drug development in chronic pain.^27^ Although pain reduction is the hallmark of successful treatment, symptoms (including pain) are often poor indicators of pathophysiology. When sufficient knowledge about pathophysiological mechanisms of disease exists, treatments can be developed that specifically target them.^35^ Pain reports are also highly variable within and across individuals^37,72,78^ and highly sensitive to social and contextual factors, which are usually independent of pathophysiology^36,44,55,56,64,81^ but related to placebo responses.^81^ These factors add substantial noise to clinical trials,^29,92^ resulting in increasing numbers of failed trials.^79^ There is a need to develop, test, and validate measures of pain-related pathophysiology in humans^1,6,12,13,22,23,25,30,40–42,50,51,53,60,61,76^ that may be used as complementary measures of interest in clinical trials. Such measures are not intended to replace pain reports^23,77^ but rather serve as physiological markers useful to track different outcomes.^25,57,74^ For example, physiological markers may be useful to confirm expected pharmacological effects on the physiological processes they are intended to target and such results can then be used to make early stop/go decisions in clinical trials.^25^

Functional magnetic resonance imaging (fMRI) could be a useful tool for understanding the neurophysiological processes that accompany chronic pain and developing biomarkers for nociceptive, cognitive/emotional, and social aspects of pain.^7,11,29,31,34,40,49,50,59,61,62,67,68^ Prior knowledge about the functional specialization of brain circuits and their alteration in pain patients complement pain report by adding a neurophysiological dimension. However, standard fMRI maps of regional brain activity are neither sensitive nor specific for any particular experiential category, including pain.^50,82,85,89^ Finding increased/decreased activity in any one region/circuit is insufficient foundation to infer changes in pain-related processes. To overcome this limitation, new approaches using pattern recognition algorithms can identify distributed patterns optimized for sensitivity and specificity to pain and other outcomes^85,15,20,39,42,50,51,57,75,80,88,91^. Here, we tested a brain measure, the neurologic pain signature (NPS),^82^ which was previously validated to track pain across multiple types of evoked noxious stimuli^14,38,45–47,50,54,82,88,92^ and shows no response to several classes of nonpainful aversive events in humans.^18,38,46,54,82,86,87^ The NPS is a distributed pattern that spans multiple brain regions involved in nociception and pain. It provides the weights used to calculate a weighted average that constitutes a brain-based predicted pain score. The NPS was developed to predict subjective pain in response to different intensities of noxious input and it is tailored to capture the association between increasing levels of nociceptive input to the brain and increasing pain ratings. Considering that the analgesic effects of naproxen occur, at least in part, via reducing nociceptive input to the brain as a result of its peripheral anti-inflammatory action,^21,32,43^ we a priori hypothesized that naproxen would significantly reduce NPS expression. We also tested the effects of placebo and naproxen on several “control” signatures beyond the NPS, for which we did not anticipate naproxen-related reductions. One such signature was the Stimulus Intensity–Independent Pain Signature 1 (SIIPS1^88^), a brain pattern more related to cognitive-evaluative aspects of pain that predicts pain after controlling for (1) noxious stimulus intensity and (2) NPS expression. We conceptualized the SIIPS1 as a control signature because we did not have previous evidence to hypothesize that naproxen would directly affect brain responses associated with the cognitive/evaluative aspects of pain after controlling for nociceptive-specific aspects; however, it is also plausible that the SIIPS1 could show effects of naproxen. Finally, we tested 2 non–pain-related control signatures predicting different types of negative emotional experiences (but not pain).^24,50^ The non–pain-related signatures, (1) the Picture-Induced Negative Emotion Signature (PINES)^18^ and (2) the Distress Signature,^4^ are whole-brain weighted patterns that were developed and validated to predict (1) ratings of negative emotion in response to aversive pictures (the PINES^18^) and (2) ratings of empathic distress while listening to others explaining difficult life experiences (the Distress Signature^4^). These signatures capture increasing levels of arousal and saliency during different kinds of distress but they are not correlated with nociceptive pain (including its evaluative components.^4,18^ We expected these signatures to show no response to noxious stimulation and no naproxen effects.

**Figure 1.**
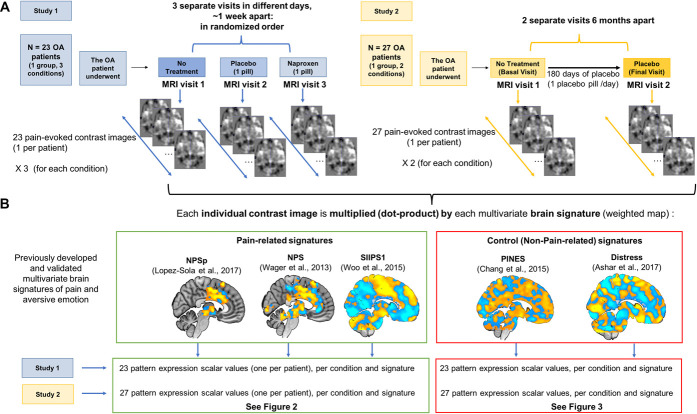
Study designs and summary of methodological approach. (A) Study designs for study 1 and study 2. The 23 patients in study 1 experienced 3 different visits in separate days: One with no treatment, one after a placebo pill, and a third after a naproxen pill (see Methods and supplementary materials for full description, http://links.lww.com/PR9/A148). The 27 patients in study 2 underwent a first visit (basal, no treatment) and a second visit (final, placebo) after receiving placebo treatment (pills) for 120 days. One contrast image representing [pain activation period (1) > rest (−1)] was obtained for each patient and each condition. (B) Summary of the methodology. Each individual contrast image was multiplied by each of the preselected, validated pain-related (NPSp, NPS, and SIIPS1) and control (emotion related, PINES and Distress) signatures (multivariate brain weighted maps that had been previously identified to maximally predict different aspects of pain or emotion in previous studies, see referenced articles). This yields one pattern response score per person per condition. NPSp, pronociceptive neurologic pain signature; NPS, neurologic pain signature; SIIPPS1, Stimulus Intensity–Independent Pain Signature 1.

This study involves a reanalysis of data from 2 previously published randomized clinical trial fMRI studies^29,59^ with the novel aims and approach of validating NPS responsiveness in 2 separate cohorts of knee osteoarthritis (OA) patients and assessing treatment responses to placebo and active pharmacological treatment (Fig. 1). We expected significant, robust NPS responses to evoked knee pain in OA patients and NPS reductions after treatment with naproxen, a nonsteroidal anti-inflammatory drug targeting inflammation through cyclooxygenase inhibition.^16,19,21,32,43^ Based on a recent meta-analysis with healthy adults,^92^ we expected the NPS to be unaffected by placebo treatment. We also anticipated a significant response on the SIIPS1 during pain in OA patients, in the same direction as in healthy adults, but not necessarily effects of naproxen. Finally, we expected the 2 emotion-related (non–pain-related) measures, the PINES and the Distress Signature, to show neither responses to painful stimulation nor naproxen or placebo effects, given previous findings showing they do not respond to painful stimulation.^18,24,88^

## 2. Methods

Figure 1 summarizes study design and methodological approach. We reanalyzed data from 2 previously published clinical trial studies^29,59^ registered in the European Clinical Trials Database EudraCT (study 1: EudraCT Number 2008-004501-33, and study 2: EudraCT Number 2009-017468) and also in one case (study 2) in ClinicalTrials.gov (Identifier: NCT01226615) to test an entirely new hypothesis not contemplated by the original clinical trials, for which study hypotheses and primary and secondary outcome measures have been published elsewhere^29,59^ (see supplementary methods for all specific method details for each study, available at http://links.lww.com/PR9/A148). Study 1 (Fig. 1) included novel analyses testing the NPS response in OA patients during knee pain and the effects of both a conventional anti-inflammatory agent and nociceptive-unspecific placebo treatment in a single cohort of 23 knee OA chronic pain patients (3 study visits in a randomized order, within-subjects design; mean age 64 ± 7.1 years, 80% female, all white Caucasian). Functional magnetic resonance imaging results from a previously published double-blind, randomized, placebo-controlled clinical trial were used for this purpose.^29^ Study 1 was based on a within-person crossover design, in which each patient participated in 3 separate sessions in different days in a randomized order, including a placebo session, a naproxen session, and a no treatment session. The study specifically tested the effects of a single oral administration of naproxen on brain responses to painful pressure stimulation in patients with knee OA, at a dose previously shown to reduce spinal sensitization.^16,58^ We hypothesized that naproxen would reduce activity in the NPS when contrasted with placebo because of its well-established analgesic effect and clear anti-inflammatory mechanism of action.

In study 2 (Fig. 1, *N* = 27, one patient cohort, within-subjects design with 2 study visits [baseline, no treatment, and placebo] after 120 days of receiving a placebo; 65.6 ± 6.2 years, 70.4% female, all white Caucasian), data from the placebo arm from an additional neuroimaging clinical trial was used to replicate the findings on NPS responses in OA patients during knee pain and test the effects of extended placebo treatment. The placebo arm from the neuroimaging clinical trial in study 2 involved 2 visits: visit 1, before placebo administration; and visit 2, after 120 days of double-blind placebo administration (for each patient randomized to the placebo arm). Study 1 and study 2 were conducted at the Hospital del Mar, Barcelona.^29,59^ The specific clinical characteristics of the patient samples and experimental procedures for each study are thoroughly described in the supplementary materials and have been previously published in detail. We include the Statistical Analysis Plan for study 1 and study 2 and the respective prior publications as supplementary materials (available at http://links.lww.com/PR9/A148).

Here, we provide a summary of the common experimental details across both studies. A detailed explanation of common experimental details can be found in the supplementary materials (available at http://links.lww.com/PR9/A148).

### 2.1. Functional magnetic resonance imaging task and painful stimuli

The same experimental paradigm was used in the scanner for both studies. The task consisted of a 6-minute sequence alternating 11 baseline “rest” periods of 20 seconds (plus a final baseline “rest” period of 30 seconds) and 11 painful stimulation periods of 10 seconds (see the detailed information in the supplementary materials, available at http://links.lww.com/PR9/A148). Immediately after the end of the MRI sequence, each subject was asked to rate the subjective pain perceived during the entire fMRI sequence using a verbally administered numerical rating scale (NRS) ranging from 0 (“no pain”) to 10 (“extreme pain”).^63^

### 2.2. Functional magnetic resonance imaging preprocessing and single-subject, first-level neuroimaging analysis

Because of strict word count limit, this section is fully described in the supplementary materials (available at http://links.lww.com/PR9/A148). In brief, fMRI time series for each study were preprocessed and analyzed using Statistical Parametric Mapping (SPM8) software, Welcome Department of Imaging Neuroscience, running on Matlab 7.1. Note that the processing and first-level model code is unchanged in SM12, and we confirmed that NPS responses did not differ as a function of whether contrast images were generated using SPM8 or SPM12.

Images were realigned to the first volume of the time series, co-registered and normalized to the Montreal Neurological Institute-space provided by SPM (voxel size = 3 × 3 × 3 mm^3^) and smoothed with a full width at half maximum Gaussian kernel of 8 mm. We provide a detailed description of our motion analyses and lack of correlation between motion parameters and NPS expression in the supplementary materials (available at http://links.lww.com/PR9/A148). In brief, we verified that the included patients had head displacements of less than 2 mm translation and 2° rotation, and for both studies, we computed mean framewise head displacement for each patient and condition following previously published methods.^66^

Consistent with previous studies,^5,29,48,59,65,82^ single-subject GLM first-level analyses in SPM included a regressor modelling pain epochs with a duration of 16 seconds, which is somewhat longer than the 10-second stimulus duration. This is advantageous for pain because previous studies have found that painful stimulation elicits fMRI activity for an extended period, and models with an extended epoch provide better fits to the data.^5,29,48,59,65,82^ This analysis also reproduces the same single-subject, first-level analysis approach presented in the clinical trial studies, which further allows for comparability between the studies.^29,59^

### 2.3. Brain signatures

Information regarding the procedure to compute signature pattern expression is described in the supplementary materials (available at http://links.lww.com/PR9/A148). The NPS includes voxel weights in an a priori defined mask of brain regions that were significantly related to the term “pain” in the Neurosynth meta-analytic database (http://neurosynth.org/); see Ref. 87 for a detailed description. Data outside this mask did not contribute to the pattern expression value. For this analysis, we used a previously defined NPS component, the “pronociceptive NPS” (NPSp), which comprised regions likely to be related to nociceptive pain (associated with pain-evoked activation in the NPS).^24,52^ In this subset of regions, which comprises most of the regions in the NPS, activity increases with increasing intensity of the noxious stimulus. These regions include the major targets of ascending nociceptive afferents, including the thalamus, secondary somatosensory regions (SI/SII), posterior, mid, and anterior insula and adjacent opercula, midbrain, dorsal anterior cingulate cortex, inferior frontal gyrus, and amygdala (Fig. 1). The SIIPS1, PINES, and Distress Signature are whole-brain weighted patterns identified using machine learning techniques. The SIIPS1 was optimized to predict pain ratings in response to acute painful stimulation after controlling for stimulus intensity and NPS expression. The PINES was optimized to predict the intensity of negative emotion ratings in response to aversive images and was shown to be unresponsive to physical pain. The Distress Signature was optimized to predict moment-by-moment experienced distress while individuals listened to true biographies describing human suffering.^4^ All these signatures were validated in independent test samples that were not included in signature training analyses. Information regarding the linear mixed effects models and planned contrasts run in this study is detailed in the supplementary materials (available at http://links.lww.com/PR9/A148). Because we had strongly directional a priori hypotheses about standard planned comparisons (drug < placebo) for the NPS and NPSp signatures, statistical tests were performed on a one-tail basis.^70,83,84^

## 3. Results

### 3.1. Pain signatures respond to evoked knee pain in osteoarthritis patients and are insensitive to placebo

#### 3.1.1. Pronociceptive neurologic pain signature and neurologic pain signature specifically respond to naproxen

**Figure 2.**
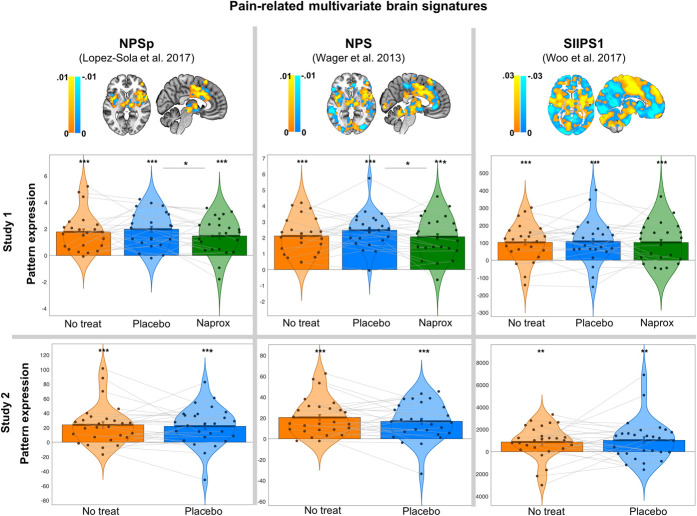
Pain-related multivariate signatures (previously published and validated) and signature response (dot-product pattern expression) for each signature, for each study and condition. The top row shows a graphic representation of the signature brain weighted maps for reference (and the original publications): the NPSp, the NPS, and the SIIPS1. Individual dots represent signature responses (dot-product pattern expression) for each OA patient in each study (2 separate cohorts, 23 patients in study 1 and 27 patients in study 2). Bars around the mean represent within-person SE bars (Loftus and Masson, 1994). ***P < 0.001, **P < 0.01, *P < 0.05. NPSp, pronociceptive neurologic pain signature; NPS, neurologic pain signature; SIIPPS, Stimulus Intensity–Independent Pain Signature 1.

We observed robust NPSp, NPS, and SIIPS1 responses during painful pressure stimulation applied to the medial articular interline of the patients' most affected knee in 2 separate randomized clinical trials (Table 1 and Fig. 2, “no treatment” condition, NPSp study 1: *t* = 5.93, Cohen *d* = 1.24, *P* < 0.001; NPSp study 2: *t* = 4.49, *d* = 0.86, *P* < 0.001; NPS study 1: *t* = 8.88, *d* = 1.85, *P* < 0.001; NPS study 2: *t* = 6.06, *d* = 1.17, *P* < 0.001; SIIPS1 study 1: *t* = 4.47, *d* = 0.93, *P* < 0.001; SIIPS1 study 2: *t* = 3.04, *d* = 0.59, *P* = 0.005). The NPSp, NPS, and SIIPS1 were reliably activated in response to knee pain in OA across both studies (mean effect size for NPSp: *d* = 1.05, mean effect size for NPS: *d* = 1.51, mean effect size for SIIPS1: *d* = 0.76; all *P*'s < 0.001). Neither one dose of placebo (study 1) nor 120 days of placebo (study 2) were associated with reductions in any of the 3 pain-specific signatures: NPSp (Table 1 and Fig. 2; study 1: *t* = −0.26, *P* = 0.54; study 2: *t* = 0.33, *P* = 0.74), NPS (study 1: *t* = −1.62, *P* = 0.13; study 2: *t* = 0.98, *P* = 0.33), or SIIPS1 (study 1: *t* = −0.33, *P* = 0.74; study 2: *t* = −0.36, *P* = 0.72) responses.

**Table 1:** Summary of signature responses for each study, condition and signature.

Signature	Condition	Study 1, mean (SD)	Study 2, mean (SD)
NPS	No treatment	2.11 (1.14)	20.43 (17.51)
	Placebo	2.47 (1.12)	16.74 (17.86)
	Naproxen	2.05 (1.29)	—
NPSp	No treatment	1.78 (1.43)	23.83 (26.58)
	Placebo	1.97 (1.32)	21.92 (26.48)
	Naproxen	1.46 (1.35)	—
SIIPS1	No treatment	102.17 (109.57)	864.1 (1473.30)
	Placebo	107.52 (123.96)	1024.5 (1838.50)
	Naproxen	101.99 (114.72)	—
PINES	No treatment	−0.008 (0.082)	−1.01 (2.01)
	Placebo	0.006 (0.089)	−0.31 (1.42)
	Naproxen	−0.002 (0.097)	—
Distress	No treatment	0.031 (0.26)	−0.532 (5.49)
	Placebo	−0.085 (0.26)	−0.434 (5.39)
	Naproxen	−0.037 (0.24)	—

Group mean and SD measures for each signature and condition are shown.

NPSp, pronociceptive neurologic pain signature; NPS, neurologic pain signature; PINES, Picture-Induced Negative Emotion Signature; SIIPPS, Stimulus Intensity–Independent Pain Signature.

In study 1, patients were exposed to a single dose of naproxen or placebo in a double-blind fashion. The 3 pain-related signatures, ie, NPSp, NPS and SIIPS1, were strongly activated during pain for the naproxen condition (NPSp: *t* = 5.18, *P* < 0.001; NPS: *t* = 7.65, *d* = 1.59, *P* < 0.001, and SIIPS1: *t* = 4.26, *P* < 0.001). As shown in Figure 2, a single dose of naproxen significantly reduced NPSp and NPS responses compared with placebo (NPSp: *t* = −2.13, *d* = 0.38, *P* = 0.02; NPS: *t* = −1.90, *d* = 0.34, *P* = 0.03) with a small-to-medium effect size. As anticipated, naproxen did not have an effect on the SIIPS1 pattern (SIIPS1: *t* = 0.21, *P* = 0.83).

Although we had planned a priori contrasts of interest as in previous work^26,70,82–84^ and given the relatively small patient samples in each study, we also ran, for completeness, a linear mixed effects repeated-measures analysis including treatment (categorical factor including the within-subject randomized conditions no treatment, placebo, and naproxen in study 1) as the predictive factor, and NPSp (model 1), NPS (model 2), and SIIPS1 (model 3) responses as the dependent variables in separate models. We found that treatment was a significant predictor (*F* = 2.79, *P* = 0.03) of NPS responses and did not reach significance when predicting NPSp responses (*F* = 1.96, *P* = 0.07). Pairwise comparisons naproxen < placebo were significant in both models (NPS *P* = 0.03 and NPSp *P* = 0.03). We did not find a significant effect of treatment on SIIPS1 (*F* = 0.53, *P* = 0.95). We did not find a drug < no treatment effect on any of the pain-related signatures in this study (all *P*'s > 0.1). This may have been because of a lack of sufficient statistical power to detect the difference given a relatively small and variable patient sample. We also checked the effects of age and gender for both studies and found no effects of age or gender for any of the analyses (all *P*'s > 0.2).

### 3.2. Emotion-related signatures do not respond to evoked knee pain in osteoarthritis patients and are insensitive to placebo and naproxen

**Figure 3.**
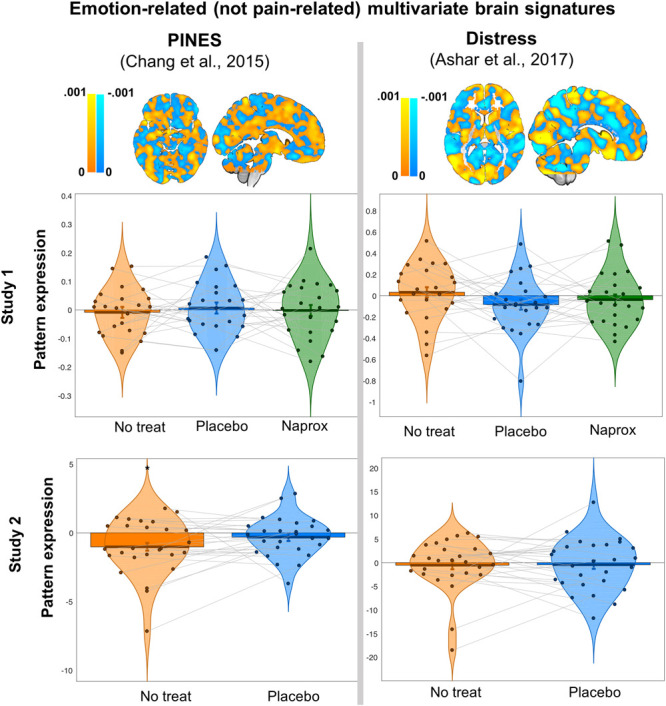
Pain-related multivariate signatures (previously published and validated) and signature response (dot-product pattern expression) for each signature, for each study and condition. The top row shows a graphic representation of the signature brain weighted maps for reference (and the original publications): the PINES (Picture-Induced Negative Emotion Signature) and Distress Signature. Individual dots represent signature responses (dot-product pattern expression) for each OA patient in each study (2 separate cohorts, 23 patients in study 1 and 27 patients in study 2). Bars around the mean represent within-person SE bars (Loftus and Masson, 1994). *P < 0.05. OA, osteoarthritis.

As anticipated, neither the PINES nor the Distress Signature were significantly positively expressed during pain for any study or group (all *P*'s > 0.1, with the exception of the PINES, which was negatively expressed—deactivated—during pain for the no treatment condition in study 2; *t* = −2.6, *d* = −0.5, *P* = 0.01; Table 1 and Fig. 3). This finding shows specificity of the PINES and Distress Signature, ie, these emotion signatures do not respond to pain in OA. Between-group effects are not meaningful when the signatures are not significantly expressed because they do not track the psychological experience they were developed to track. However, for completion, we run the preplanned contrasts of interest and the linear mixed effects repeated-measures analysis with the emotion-related control signatures.

Neither a single dose (study 1) nor 120 days of placebo (study 2) were associated with reductions in any emotion-related control signature: PINES (study 1: *t* = −0.69, *P* = 0.49; study 2: *t* = −1.70, *P* = 0.12), Distress Signature (study 1: *t* = 1.62, *P* = 0.12; study 2: *t* = −0.08, *P* = 0.94) responses (Table 1 and Fig. 3).

In study 1, patients were exposed to a single dose of naproxen or placebo in a double-blind fashion. None of the emotion-related control signatures were activated during the pain naproxen condition (PINES: *t* = −0.09, *P* = 0.93; distress: *t* = −0.73, *P* = 0.47; Table 1 and Fig. 3). As shown in Figure 3, a single dose of naproxen did not have an effect on the PINES nor on the Distress Signature compared with placebo (PINES: *t* = 0.29, *P* = 0.78; distress: *t* = −0.69, *P* = 0.49).

We also ran a linear mixed effects repeated-measures analysis including treatment (categorical factor including the within-subject randomized conditions like no treatment, placebo, and naproxen in study 1) as the predictive factor and PINES (model 1) and Distress Signature (model 2) responses as the dependent variables in separate models. We found that treatment was not a significant predictor neither of PINES (*F* = 0.20, *P* = 0.82) nor of Distress Signature responses (*F* = 1.4, *P* = 0.26).

### 3.3. Voxel-wise whole brain comparisons

For completeness, we performed a voxel-wise whole brain analysis for each of our 2 planned contrasts of interest (no treatment vs placebo for study 1 and for study 2, and placebo vs naproxen for study 1). These analyses tested for significant effects in brain regions not included in the NPS or NPSp. We found no significant differences at *P* < 0.05; false discovery rate corrected for multiple comparisons (corrected within either whole brain or gray matter only).

### 3.4. Effects of placebo interventions and naproxen on pain ratings

Table 2 shows pain ratings for each study and condition (mean and SD). Neither of the placebo interventions presented in this study modified subjective pain perception (study 1: *t* = 0.71, *d* = 0.14, SEM = 0.31, *P* = 0.49; study 2: *t* = 0.67, *d* = 0.12, SEM = 0.44, *P* = 0.51). Single-dose naproxen significantly attenuated pain ratings (vs single-dose placebo, *t* = 2.13, *d* = 0.45, SEM = 0.28, *P* = 0.02). For completeness, we also ran a linear mixed effects repeated-measures analysis including treatment (categorical factor including the within-subject randomized conditions like no treatment, placebo, and naproxen in study 1) as the predictive factor and pain ratings as the dependent variable. We found that treatment was a significant predictor (*F* = 4.05, *P* = 0.01) of pain ratings, with significant pairwise comparisons for naproxen < no treatment (*t* = 3.04, *d* = 0.64, SEM = 0.27, *P* = 0.004) and naproxen < placebo (*t* = 2.13, *d* = 0.45, SEM = 0.28, *P* = 0.02).

**Table 2:** Pain ratings for each study and condition and statistical comparison of pain ratings across conditions for each study.

	Study 1	Study 2
No treatment	Mean (SD) = 7.00 (1.00)	Mean (SD) = 7.19 (1.07)
Placebo	Mean (SD) = 6.78 (1.20)	Mean (SD) = 6.89 (2.39)
Naproxen	Mean (SD) = 6.17 (1.11)	—
Placebo < no treatment	*t*(22) = 0.71, *d* = 0.15, *P* = 0.49	*t*(26) = 0.67, *d* = 0.13, *P* = 0.51
Naproxen < placebo	*t*(22) = 2.13, *d* = 0.45, *P* = 0.02	—

### 3.5. Correlation between neurologic pain signature and pain ratings

There were no significant between-person (individual differences) correlations between NPS or NPSp responses and subjective pain ratings (for neither study group, all *P*'s > 0.2). Table 2 shows the summary of the effects of condition on pain ratings.

## 4. Discussion

The NPS and its specific pronociceptive component, the NPSp, were activated in response to knee pain in OA across studies and did not respond to 2 different kinds of placebo interventions. Naproxen, a commonly prescribed anti-inflammatory drug for chronic OA pain, reduced NPS and NPSp responses beyond placebo, in agreement with reductions in pain ratings. We found no effect of placebo or naproxen on the SIIPS1, which specifically tracks pain after controlling for stimulus intensity. We also checked responses in 2 emotion-related brain markers that have shown high sensitivity and specificity for negative emotions in different contexts unrelated to pain. We found no significant pain-related response of these markers and no significant effects of placebo or naproxen, hence providing further proof of specificity to the NPS and NPSp findings. This study provides initial proof of concept that fMRI-based measures validated for nociceptive pain can be sensitive to evoked knee OA pain and to active treatment. Larger samples are required to confirm and extend our results. The results add utility value to the use of neurophysiological brain markers always in combination with main outcome measures of pain and disability in clinical trials. Multivariate markers like the NPS or the NPSp can be useful particularly in the context of limited sample sizes (eg, early-stage clinical trials and most patient studies without major financial backing). Multivariate brain markers provide a set of interpretable summary measures across hundreds of thousands of brain voxels, avoiding the need to correct for multiple comparisons when validating marker performance. Developing and validating new brain markers capitalizing on pain modulation mechanisms not captured by the NPS or SIIPS1 could help identify neurophysiological effects of treatments that are unrelated to nociceptive-specific factors. The study has some limitations. First, the results for study 1 are based on 1.5-Tesla MRI scanner; second, sample sizes for both studies, particularly study 1 (*N* = 23 patients), are small, and all patients' ethnicity was white Caucasian, which accentuates the need for future replication in larger, more diverse patient samples.

The lack of placebo effect on NPS, NPSp, or SIIPS1 responses suggests that placebo is not targeting the neurophysiological process captured by the NPS—ie, nociceptive processing at the brain level—or the SIIPS1—cognitive-evaluative brain processes predicting pain after controlling for stimulus intensity and NPS—even under a regime involving 120 days of placebo administration. However, placebo treatment did not significantly affect pain in these samples, so it is possible that a more “powerful placebo” would have shown an effect on the NPS or SIIPS1. Previous studies that did show placebo effects on pain also showed null or very small effects on the NPS,^82,92^ suggesting that even effective placebo manipulations may have much smaller effects on the NPS than they do on pain reports.

There are multiple other brain, spinal, and peripheral mechanisms that contribute to modulating pain that are not represented neither in the NPS nor directly in the SIIPS1. For example, the NPS does not include (or only partially includes) contributions from the lateral and medial prefrontal cortex, ventral striatum, and some brainstem regions. These regions modulate pain responses and have been associated with transitions from acute to chronic pain states^2,3,8,10,17,31,52,73,87,88^ and represent other potential neurophysiological treatment targets. Thus, multivariate markers like the NPS or the NPSp can be useful, particularly for limited sample sizes (eg, early-stage clinical trials and most patient studies without major financial backing).

The NPS was developed in young healthy adults during acute thermal pain in the forearm^82^; in this study, it is tested in older chronic pain patients with pain in the affected knee and during painful knee pressure. Previous literature provides robust evidence for acute knee pain–evoked activation in OA patients in regions overlapping with the NPS marker, including somatosensory cortices, insula, basal ganglia, thalamus, midbrain, anterior cingulate cortex, and amygdala.^9,33,59,62,71,90^ As anticipated, the NPS showed good generalizability to this clinical population, to a different pain modality and when applied to a clinically affected site in 2 different OA patient samples. We observed a difference in absolute NPS scale between study 1 and study 2. Multiple factors influencing the absolute scale of the NPS response usually differ across studies, including MRI field strength, different experimental designs, voxel size, and first-level contrast (beta) image weights.^54^ Study 1 and study 2 differed in MRI field strength, voxel sizes, and first-level contrast image weights, which explains absolute scale differences. Currently, BOLD fMRI responses are not considered “quantitative” in the sense that one cannot compare absolute quantities across studies. The NPS can be used to quantify effect sizes for relative comparisons within a study (eg, drug vs placebo), but establishing absolute quantitative values across studies remains a challenge. We did not attempt to equate the absolute scale of the NPS response across studies because the reported within-study comparisons are unaffected by scale issues.

Confirming our initial hypothesis, we found a reduction of NPS and NPSp responses by naproxen vs placebo. The reduction became numerically stronger—numerically larger in magnitude—when the NPS response was tested specifically on nociceptive regions (NPSp),^24,50^ which agrees with the observed reduction in subjective pain reports after naproxen. NPS reductions after naproxen—an anti-inflammatory drug with previously identified nociceptive effects at the peripheral and central nervous system levels^16,19,21,32,43,58^—argues in favor of the NPS and NPSp as good summary measures of drug effects (vs placebo) on nociceptive processing in the human brain. NPS and NPSp reductions during naproxen vs placebo align with findings from a neuroimaging placebo-controlled trial testing naproxen effects on brain activity.^71^ The study showed that naproxen reduced brain activation over placebo in bilateral primary somatosensory cortex, thalamus, and amygdala: all regions included in the NPS/NPSp. In the same line, in the previously published clinical trial results from study 1,^29^ our group found preliminary (uncorrected) effects of naproxen in the second somatosensory cortex, bilateral insula, basal ganglia, ACC, and amygdala. Although these studies provide detailed insight about the brain regions that were modulated by naproxen over placebo, they lacked sufficient statistical power to survive correction for multiple comparisons.

The current results regarding naproxen effects on the NPS and NPSp require replication in larger samples and using different naproxen doses, particularly because the effects were specific to the comparison naproxen < placebo and were small to medium in effect size. The rationale for using naproxen to test its effects on the NPS over placebo was based on its well-established antinociceptive action,^16,19,21,32,43^ which was deemed optimal to test the hypothesis that a drug with known antinociceptive effects should significantly reduce NPS beyond the nociceptive-unspecific effects of placebo. That said, the study does not provide data for comparison with healthy controls, other forms of knee-free chronic pain patients or, importantly, other forms of treatment. Future studies should compare the effects of naproxen with other commonly used pharmacological, psychological, and physical therapies for OA. Neurophysiological biomarkers in combination with conventional outcome measures in clinical trials for pain may show potential for helping our understanding of the effects of different treatments on previously characterized and validated neurophysiological components of pain. Future studies may successfully develop new markers of spontaneous pain, which may complement information summarized by the NPS/NPSp by relying on partially nonoverlapping brain circuits.^7,9,28,62^ By testing new brain markers that show sensitivity and specificity for different types of human pain experiences, acute and chronic, evoked and spontaneous,^69,77,85^ involving different pain modalities and in different body locations, we can start generating more clinically translatable imaging models with the potential to optimize current and future treatments.

## Disclosures

The authors have no conflicts of interest to declare.

## Appendix A. Supplemental digital content

Supplemental digital content associated with this article can be found online at http://links.lww.com/PR9/A148.

## Supplementary Material

SUPPLEMENTARY MATERIAL
