# Supplementary material for: The neurologic pain signature responds to nonsteroidal anti-inflammatory treatment vs placebo in knee osteoarthritis
Source: Pain Rep. 2022 Feb 16;7(2):e986. doi: 10.1097/PR9.0000000000000986 (PMC8853614; doi:10.1097/PR9.0000000000000986)
Supplement: SUPPLEMENTARY MATERIAL [file painreports-7-e986-s001.pdf]

SUPPLEMENTARY METHODS AND REFERENCES

Specific experimental details for each study

Study 1

Study 1 is a placebo-controlled, double-blind, randomized, three-session crossover study (see all details in [3]). Patients were randomly assigned to four possible randomization schedules (A/C/B; B/C/A; C/A/B; C/B/A. A: Naproxen 500 mg, B: Placebo, C: No Treatment). Independent pharmacists dispensed the pills to the investigator according to the randomization list, and both patients and investigators remained blind to treatment. Both Naproxen and Placebo were administered in capsule form and were identical in appearance and administration method. The trial was registered at the European Clinical Trials Database, "EudraCT Number 2008-004501-33". Patients received oral Naproxen 500 mg (nonsodium form of Naproxen, Naproxyn EC, Roche Products Ltd.), Placebo, or No Treatment in each of the 3 separate sessions and were dosed in the unit. fMRI assessments were fixed to start at 4 h post-dosing and stop by 5 h post-dosing (based on the peak plasma concentration of Naproxen at 2–4 h, see [3].

Patients. The study took place at the Hospital del Mar in Barcelona in collaboration with additional primary healthcare centers. Full recruitment and retention details are provided in the original report by [3]. In brief, written informed consent was obtained from all patients and the study was approved by the local Ethics Committee (Clinical Research Ethical Committee- Institut Municipal d'Assistència Sanitària, Barcelona), and conducted in compliance with the Code of Ethics of the World Medical Association (Declaration of Helsinki). A total of 27 patients with OA were randomized. There were no treatment-related adverse events. Randomized patients had a mean age of 64 ± 7.1, range 52 to 79 years, 80% female, and were all white Caucasian. Patients had a baseline pain (subjective pain experienced in the affected OA knee at the screening visit without receiving any painful stimulation) of 6.2 ± 1.1 (in a NRS from 0 "No pain" to 10 "Extreme pain"), BMI of 29.9 ± 3.2 (24.9–35.3), and 8% of them were depressed. Out of the 27 patients, 23 were included in the analysis after verifying the acceptability of fMRI data quality [3]. Four patients were excluded from analyses due to cavernoma affecting fMRI signal (1), poor image quality (2), randomization error (1).

A patient was eligible for inclusion if all of the following criteria applied: (i) a diagnosis of OA and suitable for the study as determined by a responsible physician, based on a medical evaluation including medical history, physical examination, and cardiac monitoring; (ii) to have a radiological and clinical diagnosis of OA based upon the American College of Rheumatology (ACR) criteria (1) affecting at least one knee for a minimum of 3 months in symptom duration prior to screening. The symptoms should be significantly worse in one knee if both knees were affected; (iii) male or female ≥ 45 years of age; (iv) a minimum and a maximum of 4 and 8 out of 10 on the numerical rating scales (NRS) at screening referring "pain at present" and/or a requirement for the use of an analgesic for the pain in the OA knee for most days during the previous 3 months. In addition, baseline pain must be stable for at least 72 hours prior to session 1; (v) a non-childbearing potential females; (vi) body weight <120 kg and Body Mass Index (BMI) within the range 15 – 35 kg/m^2.

Exclusion criteria were: (i) to have a positive pre-study drug/alcohol screen or history of alcohol abuse within 6 months of the study; (ii) use of prescription or non-prescription drugs, including vitamins, herbal and dietary supplements within 7 days or 5 half-lives prior to the first dose of study medication; (iii) history or presence of gastro-intestinal, hepatic or renal disease or other condition known to interfere with the absorption, distribution, metabolism or excretion of drugs; (iv) subject was taken any drug known to induce or inhibit hepatic drug metabolism in the 15 days prior to the screening visit at

the start of the study; (v) subjects who have asthma or a history of asthma, (vii) subjects smoking more than 20 cigarettes per day; (viii) secondary causes of arthritis of the knee; (ix) had lower extremity surgery within 6 months prior to screening or scheduled for surgery of any kind during the study period in the affected knee; (x) significant prior injury to the index knee within 12 months prior to screening; (xi) disease of the spine or other lower extremity joints to affect the index knee or any other musculoskeletal or arthritic condition that may affect the interpretation of clinical efficacy and/or safety data; (xii) use of any analgesic, cyclooxygenase-2 (COX-2) inhibitor or nonsteroidal anti-inflammatory drugs (NSAIDs), other than study defined rescue therapy within 5× half-life prior to the first dosing day or during the study; (xiii) corticosteroid use prior to baseline; (xiv) received hyaluronan injections into index knee within the previous six months prior to baseline; (xv) initiation of or change to an established physiotherapy program within 2 weeks prior to baseline or during the study period.
During the baseline clinical assessment, patients rated the severity of pain for left and right knees on a numerical rating scale (NRS) (0: "No pain"; 10: "Extreme pain"). The most severe knee was used in each session.

fMRI parameters. A 1.5 T Signa Excite system (General Electric) equipped with an 8-channel, phased-array head coil and single-shot echo planar imaging software was used. Functional sequences consisted of gradient recalled acquisition in the steady state (time of repetition [TR], 2000 ms; time of echo [TE], 50 ms; pulse angle, 900; field of view [FOV], 24 cm; 64 x 64-pixel matrix; slice thickness, 4 mm (inter-slice gap, 1.5 mm)). Twenty-two interleaved slices, parallel to the anterior-posterior commissure line, were acquired to cover the whole-brain. The acquisitions were preceded by 4 additional dummy images allowing the fMRI signal to reach equilibrium.

Study 2
In order to further test NPS responses to pressure pain and the presence or absence of Placebo effects, we re-analyzed data corresponding to the Placebo arm (before [no treatment] and after 120 days of Placebo) of a previously published [10] randomized, double-blind, placebo-controlled trial (ClinicalTrials.gov. Identifier: NCT01226615) conducted at Hospital del Mar, Barcelona, Spain.

Patients. N = 32 patients were scanned, and 27 of these passed fMRI quality control and were included in the final analysis [10]. Patients' mean age was 65.6 ± 6.2 years, 19 were female, all were white Caucasian, had a knee pain severity at baseline (NRS) of 6.4 ± 1.3 (in a NRS from 0 "No pain" to 10 "Extreme pain"), 21 patients had radiological grade II whereas 3 were classified as radiological grade III. 11 had regular use of NSAIDs to control clinical pain. In brief, the study was approved by the local Ethics Committee (Clinical Research Ethical Committee-Institut Municipal d'Assistència Sanitària (CEIC-IMAS)) and run in compliance with the World Medical Association's Code of Ethics (Declaration of Helsinki), with written informed consent from all patients.
Eligibility criteria. A subject was eligible for inclusion when all of the following criteria applied: (i) male or female with age between 40 and 75 years; (ii) diagnosis of primary osteoarthritis and suitability for the study as determined by the responsible rheumatologist, based on a comprehensive medical evaluation; (iii) radiological and clinical osteoarthritis based on the American College of Rheumatology (ACR) criteria;[1] (iv) osteoarthritis radiological grade II or III;[2] (v) stable knee symptoms for at least 1 month prior to screening; and (v) a minimum knee pain severity of 5 points on the 11-point numerical rating scales (NRS) at baseline.
Exclusion criteria: (i) clinical evidence or history of drug/alcohol addiction; (ii) previous adverse effect to CS; (iii) relevant, non-controlled medical or psychiatric disease; (iv) formal MRI contraindication; (v) severe pain in other joints; (vi) inflammatory or systemic diseases with potential repercussion on joints; (vii) secondary causes of arthritis of the knee; (viii) the use of any analgesic, cyclooxygenase-2 (COX-2) inhibitor or nonsteroidal anti-inflammatory drugs

(NSAIDs) within seven days prior to inclusion or during the study; (ix) systemic use or local corticosteroid injection three months prior study and during the study; (x) use of CS, diacereine, glucosamine or other symptomatic slow acting drugs for osteoarthritis three months prior the study; (xi) hyaluronan injections into the index knee within the previous six months prior to the study; (xii) subjects smoking over 20 cigarettes a day; (xv) initiation or change of a physiotherapy program in the 2 weeks prior to screening or during the study period; and (xvi) females of childbearing potential.

Following screening, eligible subjects underwent a 2-week medication washout prior to the first fMRI assessment. Placebo treatment consisted of 2 hard gelatin yellow capsules taken together once a day during a meal. The capsule contained microcrystalline cellulose as a non-active excipient. During the baseline clinical assessment, patients rated pain severity using an 11-point NRS. Again, the most severe knee was used during the fMRI assessment.

<u>fMRI parameters.</u> A 3T MRI scanner (Achieva X series, Philips Medical Systems, Eindhoven, The Netherlands) equipped with an eight-channel phased-array head coil and single-shot echo planar imaging (EPI) software was used. Functional sequences consisted of gradient recalled acquisition in the steady state (time of repetition [TR], 2000ms; time of echo [TE], 35ms; pulse angle, 90°; field of view [FOV], 23cm; 96×69-pixel matrix; slice thickness, 4mm (plus inter-slice gap, 1 mm)). Twenty-two interleaved slices, parallel to the anterior-posterior commissure line, were acquired to cover the brain. The pain functional time series consisted of 180 consecutive volumes over 6 minutes. The acquisitions were preceded by 4 additional dummy volumes to allow MRI signal to reach equilibrium.

<u>Here we provide a description of the common experimental details across both studies.</u>

<u>fMRI task and painful stimuli.</u> The same experimental paradigm was used in the scanner for both studies. The task consisted of a 6 min sequence alternating 11 baseline "rest" periods of 20 s (plus a final baseline "rest" period of 30 s) and 11 painful stimulation periods of 10 s. Pressure painful stimulation was applied using an MRI-compatible algometer developed in-house, with a pressure surface of 1 square cm [3]. Patients received pressure stimulation on the medial articular interline of the selected knee (most painful knee) at the most tender point in each subject with the knee in the position of 60° flexion. The tender point was established by palpation and marked using a permanent felt-tip pen. Pressure intensity to be applied during fMRI acquisition was calibrated to evoke pain between 5 and 7 on an 11-point NRS. Such a pressure adjustment was performed before dosing in each fMRI session day in study 1. There were no significant differences in applied pressure intensity between the No Treatment, Placebo, and Naproxen visits (ANOVA results, $F = 0.38$ and $p = 0.687$; mean pressure intensity for Naproxen was (SD): 2.6 Kg (1.0 Kg); Mean pressure intensity for Placebo was 2.5 Kg (0.8 Kg)), and mean pressure intensity for No Treatment was 2.6 Kg (0.9 Kg). In Study 2, the same pressure intensity was applied during the fMRI visits for the No Treatment and Placebo conditions (mean ± SD applied pressure was 2.5 kg ± 1.1 Kg). Each subject was asked to rate the subjective pain perceived during the entire fMRI sequence immediately after fMRI acquisition using a NRS.

<u>Image preprocessing and motion analyses.</u> fMRI time series for each study were preprocessed and analyzed using Statistical Parametric Mapping (SPM8) software, Welcome Department of Imaging Neuroscience, running on Matlab 7.1. Note that the processing and first-level model code is unchanged in SM12, and we confirmed that NPS responses did not differ as a function of whether contrast images were generated using SPM8 or SPM12.

Images were realigned to the first volume, co-registered and normalized to the Montreal Neurological Institute (MNI)-space provided in SPM8 (voxel size = 3x3x3 mm^3) and smoothed with a full width at half maximum (FWHM) Gaussian kernel of 8 mm. We verified that the included patients had head displacements of less than 2mm translation and 2º rotation. For both studies, we also computed mean framewise head displacement for each patient and condition following previously published methods [12]. In brief, motion was quantified using the realignment parameters obtained during image preprocessing, which included 3 translation and 3 rotation estimates. Average inter-frame motion measures (head position variations of each volume as compared to the previous volume) were used to capture head motion across the entire scan sequence [11,14,15]. A motion summary measure (mean framewise head displacement) that combined translations and rotations was computed in mm by adapting the formula of Van Dijk et al. [15] (for further explanations see [12], supplementary information). We ran a linear mixed effects repeated measures analysis to assess the effect of condition on mean framewise head displacement for Study 1 and showed no effect of treatment session on average framewise head displacement (F=0.47, p=0.63). Results from a paired samples t-test for Study 2 showed no effect of session (No Treatment, Placebo) on mean framewise head displacement (t=-0.93, p=0.36). Last, we performed a correlation analysis between mean framewise head displacement and NPS expression for each condition for each study and found no significant correlation for any comparison (all p's>0.1).

Signature pattern expression. For both studies the same procedure was used to compute pattern response (or 'pattern expression') values for each patient and each condition (i.e., Placebo, No Treatment and Naproxen). For each patient we computed a single scalar value representing the expression of the NPS pattern (NPS in pro-nociceptive regions, and entire NPS pattern), SIIPS1 pattern, PINES pattern or Distress pattern for the contrast images representing "pain > rest" (as explained in detail in previous articles [5–8]). The NPS includes voxel weights in an *a priori* defined mask of brain regions that were significantly related to the term "pain" in the Neurosynth meta-analytic database (http://neurosynth.org/), see [16] for a detailed description. Data outside this mask did not contribute to the pattern expression value. For this analysis, we used a previously defined NPS component, the "Pronociceptive NPS" (NPSp), which comprised regions likely to be related to nociceptive pain (associated with pain-evoked activation in the NPS) [1,8]. In this subset of regions, which comprises most of the regions in the NPS, activity increases with increasing pain. These regions include the major targets of ascending nociceptive afferents, including the thalamus, secondary somatosensory regions (SI/SII), posterior, mid and anterior insula and adjacent opercula, midbrain, dorsal anterior cingulate cortex (dACC), inferior frontal gyrus and amygdala (Figure 1). The SIIPS1, PINES, and Distress signatures are whole-brain patterns, i.e., they were developed including weights across the whole brain. For every contrast image of each patient participant, we computed the dot product of the vectorized activation contrast image (β_{map}) with the NPSp (or NPS, or SIIPS1, or PINES or Distress Signature) pattern of voxel weights ($NPS\text{-}\omega_{map}$), i.e., $\beta_{map}{}^{T} NPS\text{-}\omega_{map}$, yielding a continuous scalar value for each patient, for the [pain vs. rest] comparison.
To test for pressure pain responsiveness, drug effects, and placebo effects, we used planned contrasts specified a priori. The [Drug vs. Placebo] comparison and the [Placebo vs. No Treatment] comparison are the most widely accepted standard for testing drug effects and placebo effects, respectively. The former is the standard comparison in randomized clinical trials of pharmacotherapy [2,13], and the latter is the standard way of assessing active placebo effects (as compared with statistical artifacts or sampling biases) [4,9]. Therefore, we planned our analyses to test these two comparisons. For the sake of completeness, we ran linear mixed effects repeated measures analyses including treatment (categorical factor) as the independent variable and (a) NPSp and NPS, (b) SIIPS1, (c) PINES, (d) Distress, and (e) pain ratings as the dependent variable (in separate models) using SPSS (version 25).

Because we had strongly directional *a priori* hypotheses about these planned comparisons, statistical tests were performed one tailed.

We used one-sample t-tests to evaluate whether NPS responses to [pain vs. rest] were above zero, as pain beta maps are implicitly contrasted with the intercept (rest) in the 1st-level model. To test within-subject contrasts for [Placebo < No Treatment], and [Naproxen < Placebo], we performed paired-samples t-tests across these conditions. Matlab software (MATLAB, MathWorks, v. R2016a, https://www.mathworks.com) and CanLab customized publicly available code (https://github.com/canlab) was used for all statistical comparisons and data illustration using bar plots with 95% confidence intervals.

SUPPLEMENTARY MATERIAL REFERENCES

[1] Duff EP, Moultrie F, van der Vaart M, Goksan S, Abos A, Fitzgibbon SP, Baxter L, Wager TD, Slater R. Inferring pain experience in infants using quantitative whole-brain functional MRI signatures: a cross-sectional, observational study. Lancet Digit Health 2020;2:e458–e467.

[2] Evidence-Based Medicine Working Group. Evidence-based medicine. A new approach to teaching the practice of medicine. JAMA 1992;268:2420–2425.

[3] Giménez M, Pujol J, Ali Z, López-Solà M, Contreras-Rodríguez O, Deus J, Ortiz H, Soriano-Mas C, Llorente-Onaindia J, Monfort J. Naproxen effects on brain response to painful pressure stimulation in patients with knee osteoarthritis: a double-blind, randomized, placebo-controlled, single-dose study. J Rheumatol 2014;41:2240–2248.

[4] Kaptchuk TJ. Powerful placebo: the dark side of the randomised controlled trial. Lancet 1998;351:1722–1725.

[5] López-Solà M, Geuter S, Koban L, Coan JA, Wager TD. Brain mechanisms of social touch-induced analgesia in females. Pain 2019;160:2072–2085.

[6] López-Solà M, Koban L, Krishnan A, Wager TD. When pain really matters: A vicarious-pain brain marker tracks empathy for pain in the romantic partner. Neuropsychologia 2017. doi:10.1016/j.neuropsychologia.2017.07.012.

[7] Lopez-Sola M, Koban L, Wager TD. Transforming pain with prosocial meaning: an fMRI study. Psychosom Med 2018.

[8] López-Solà M, Woo C-W, Pujol J, Deus J, Harrison BJ, Monfort J, Wager TD. Towards a neurophysiological signature for fibromyalgia. Pain 2017;158:34–47.

[9] Meissner K, Bingel U, Colloca L, Wager TD, Watson A, Flaten MA. The placebo effect: advances from different methodological approaches. J Neurosci 2011;31:16117–16124.

[10] Monfort J, Pujol J, Contreras-Rodríguez O, Llorente-Onaindia J, López-Solà M, Blanco-Hinojo L, Vergés J, Herrero M, Sánchez L, Ortiz H, Montañés F, Deus J, Benito P. Effects of chondroitin sulfate on brain response to painful stimulation in knee osteoarthritis patients. A randomized, double-blind, placebo-controlled functional magnetic resonance imaging study. Med Clin 2017;148:539–547.

[11] Power JD, Barnes K a., Snyder AZ, Schlaggar BL, Petersen SE. Spurious but systematic correlations in functional connectivity MRI networks arise from subject motion. Neuroimage 2012;59:2142–2154.

[12] Pujol J, Macià D, Blanco-hinojo L, Martínez-vilavella G, Sunyer J, De R, Caixàs A, Martín-santos R, Deus J, Harrison BJ. NeuroImage Does motion-related brain functional connectivity re fl ect both artifacts and genuine neural activity ? Neuroimage 2014;101:87–95.

[13] Sacks H, Chalmers TC, Smith H Jr. Randomized versus historical controls for clinical trials. Am J Med 1982;72:233–240.

[14] Satterthwaite TD, Elliott M a., Gerraty RT, Ruparel K, Loughead J, Calkins ME, Eickhoff SB, Hakonarson H, Gur RC, Gur RE, Wolf DH. An improved framework for confound regression and filtering for control of motion artifact in the preprocessing of resting-state functional connectivity data. Neuroimage 2013;64:240–256.

[15] Van Dijk KR a., Sabuncu MR, Buckner RL. The influence of head motion on intrinsic functional connectivity MRI. Neuroimage 2012;59:431–438.

[16] Wager TD, Atlas LY, Lindquist M a., Roy M, Woo C-W, Kross E. An fMRI-based neurologic signature of physical pain. N Engl J Med 2013;368:1388–1397.
